# Promising Neutrophil-Associated Biomarkers in Lung Diseases of Patients with Antisynthetase Syndrome and Dermatomyositis

**DOI:** 10.1155/2022/1886083

**Published:** 2022-09-26

**Authors:** Lei Liu, Jundi Wang, Peiyu Zhang, Wenjia Sun, Xiuliang Zhu, Xiujuan Sun, Jing Xue, Huaxiang Wu

**Affiliations:** ^1^Department of Rheumatology, The Second Hospital Affiliated to Zhejiang University, College of Medicine, China; ^2^Department of Rheumatology, Affiliated Hangzhou First People's Hospital, Zhejiang University School of Medicine, China; ^3^Department of Radiology, The Second Hospital Affiliated to Zhejiang University, College of Medicine, China

## Abstract

**Objectives:**

Antisynthetase syndrome (ASS) and dermatomyositis (DM) are serious autoimmune diseases, with lungs being the most frequently involved organ and sometimes fatal. This study is aimed at clarifying the role of neutrophil-associated biological markers in suggesting ASS and DM-associated respiratory infections and interstitial lung diseases.

**Methods:**

We carried out a retrospective review of the medical records of 46 cases of ASS and DM diagnosed at the Second Hospital of Zhejiang University College of Medicine, between January 2017 and December 2020. Serum myeloperoxidase (MPO), neutrophil elastase (NE), *α*1 anti-trypsin (AAT), and interleukin-6 (IL-6) were also detected.

**Results:**

Gottron's sign is characteristic of dermatomyositis, while polyarthritis is more characteristic of ASS. Pulmonary function is worse in ASS than in DM patients. Patients with ASS and DM had abnormal lymphocyte and neutrophil counts compared to healthy subjects, but not in relation to lung function and rapid progression of interstitial lung disease (RP-ILD). Elevated serum NE, MPO, and IL-6 levels are suggestive of respiratory infections, whereas decreased circulating IL-6 is predictive of RP-ILD.

**Conclusion:**

Our study identified the neutrophil-associated biomarkers MPO, NE, and IL-6 as promising indicators with different suggestive roles in respiratory infections and interstitial lung diseases in patients with ASS and DM.

## 1. Introduction

Idiopathic inflammatory myopathy (IIM) is a rare group of heterogeneous autoimmune diseases characterized by symmetric proximal muscle weakness and myositis-specific or associated autoantibodies, with extramuscular manifestations, and usually with high morbidity and mortality [1]. Lung involvement is the most common cause of death, especially in patients with ASS and DM [2]. The lack of effective and convenient indicators for monitoring lung diseases is an important factor in the poor prognosis of this group of patients. Although a number of predictive models have been reported [3–5], they often include indicators such as pulmonary imaging changes or lung function that are not easily reproducible in a short period of time, and the pulmonary progress associated with ASS and DM are often rapid. Therefore, it is still necessary to explore novel biomarkers that can predict the patient's lung involvement. In particular, biomarkers for respiratory infections, RP-ILD, and early warning of lung function decline in patients are of great importance. Neutrophils are important innate immune cells; several studies have explored the pathogenesis of neutrophils in IIM. He and colleagues reported that acute and subacute interstitial lung diseases of DM patients group had a significantly higher proportion of neutrophils than the chronic group [6]. Very late antigen-4 expression on neutrophils might be useful for detecting the severity of lung diseases in patients with DM [7]. In addition, other specific biological forms or associated factors of neutrophils, like neutrophil extracellular traps (NETs), may also play an important role in IIM. NETs containing human cathelicidin were reported to be increased in patients with IIM and active disease and correlate with proinflammatory cytokines [8]. Peripheral NET levels are associated with calcinosis and interleukin (IL)-8 levels [9]. Data from Zimmermann et al. clarified the ability of human neutrophils to generate IL-6 [10]. In many studies on COVID-19, IL-6 has been proved to be associated with interstitial lung disease [11, 12]. AAT is a circulating serine protease inhibitor that inhibits a variety of proteases, especially NE. AAT could also regulate the expression of IL-6. Numerous studies have been conducted in structural lung diseases such as chronic obstructive pulmonary disease (COPD) and pulmonary cystic fibrosis [12]. Our study was to reveal the prognostic role of these aforementioned neutrophil-associated biomarkers in the lung diseases of ASS and DM.

## 2. Methods

### 2.1. Patients

This was a retrospective, single-center study of patients diagnosed with ASS or DM between January 2017 and December 2020 at the Second Affiliated Hospital of Zhejiang University College of Medicine. A total of 46 patients, who visited our hospital for the first time, were enrolled in this study. Serum of all patients was kept on the first day of admission. The diagnoses were made according to the 2017 European League Against Rheumatism (EULAR)/American College of Rheumatology (ACR) classification criteria for adult and juvenile idiopathic inflammatory myopathies and their major subgroups comprehensive [13]. Since this study mainly focuses on IIM-related lung diseases and we have the data of myositis-specific antibodies of all enrolled patients, we combined patients with ADM and DM as DM for further study according to the new classification system [14]. Patients who met Connor's Criteria were diagnosed as ASS and excluded from DM [15]. Data of the healthy people (healthy control, HC, *n* = 46) were from the Health Examination Center of the Second Affiliated Hospital of Zhejiang University College of Medicine. The Medical Ethics Committee of the Second Affiliated Hospital of Zhejiang University, College of Medicine, approved this study (no. 2017-113). The informed consents of patients were obtained.

### 2.2. Data Collection

We reviewed the electronic medical records of all included IIM patients and collated the basic information, clinical manifestations, treatment, laboratory results, and radiological findings. Two experienced radiologists assessed chest high-resolution computer tomography (HRCT) images. Interstitial lung disease was mainly judged according to the clinical presentation of the patient and the chest HRCT. Diagnosis of respiratory infections was based on a combination of clinical manifestations, such as cough, sputum, and/or fever, laboratory investigations, and chest HRCT. The definition of RP-ILD is progressive dyspnea occurring within one month after the onset of respiratory symptoms and/or new interstitial abnormalities on chest radiography [3, 16]. Laboratory results including myositis-specific or associated autoantibodies and pulmonary function were obtained from the hospital's electronic medical record system.

### 2.3. MPO and NE Assessment

MPO and NE originating from NETs are in the form of MPO:DNA complex and NE:DNA complex in the circulation. Therefore, MPO:DNA complex and NE:DNA complex (abbreviated as MPO and NE in the paper) were detected in this study.

### 2.4. Human MPO:DNA Complexes

Human MPO:DNA complexes were detected as previously reported [17]. High binding 96-well ELISA microplates were incubated overnight at 4°C with mouse anti-human MPO (clone 4A4; AbD Serotec) in coating buffer from the Cell Death Detection ELISA kit (cat# 11544675001; Roche). The Cell Death Detection ELISA kit is for determination of cytoplasmic histone-associated DNA fragments (mono- and oligonucleosomes) after induced cell death. We take the coating buffer and DNA-POD for detecting DNA part of MPO:DNA and NE:DNA. After blocking with 1% BSA (cat# A7906; Sigma) in PBS, the plates were incubated overnight at 4°C with 10% human plasma in blocking buffer and washed, and anti-DNA-POD (clone MCA-33; Roche) was added for 1.5 hours at room temperature. Following incubation, TMB substrate (cat# T0440; Sigma) was added, and absorbance was measured at 450 nm after addition of stop reagent (cat# S5814; Sigma).

### 2.5. Human Neutrophil Elastase (NE):DNA Complexes

Human NE:DNA complexes were detected as previously reported [17]. High binding 96-well ELISA microplates were incubated overnight at 4°C with rabbit anti-human HNE6 (cat# 481001; Calbiochem) in PBS. After blocking, the plates were incubated overnight with 10% human plasma in blocking buffer, washed, and incubated for 1 hour at room temperature with mouse anti-dsDNA mAb (clone 16-13; EMD Millipore), followed by anti-mouse IgG-HRP conjugate (cat#1706516; BioRad, Hercules, CA). Procedure was completed as for MPO:DNA complexes.

### 2.6. AAT and IL-6 Assessment

A human AAT ELISA kit was purchased from BOSTER (cat# EK1634) and was used to measure the levels of serum AAT. The detection range of the ELISA kit was 1.56 ng/mL-100 ng/mL. A human IL-6 kit was purchased from Invitrogen (cat# BMS213-2) and was used to measure the levels of serum IL-6. The detection range of the ELISA kit was 1.56 pg/mL-100 pg/mL. All assays were performed according to the manufacturer's recommendations. Cytokine concentrations were determined from standard concentrations plotted to create a standard curve.

### 2.7. Statistical Analysis

GraphPad Prism 8.0.1 (GraphPad Software, La Jolla, CA) was used for statistical analyses. Continuous variables are presented as the mean (S.D.) for a normal distribution or the median (interquartile range 25th–75th percentile) for a nonnormal distribution. Categorical variables are presented as frequencies. An independent sample *t*-test was used for pairwise comparison between groups of continuous variables with normal distribution and equal variance. The Wilcoxon rank-sum test was performed to analyse continuous data with a nonnormal distribution. Categorical variables were tested with a chi-square test. A *P* value < 0.05 was considered statistically significant.

## 3. Results

### 3.1. Demographics and Clinical Characteristics of ASS and DM Patients

All of the 46 IIM patients were included in the analysis. Demographics and clinical characteristics of ASS patients were compared with DM patients (Table 1). There were 20 patients with ASS and 26 patients with DM. The age of all the patients was 57.3 ± 10.9 years, with females accounting for the majority (71.7%). Of all the patients, twenty-two were first diagnosed with IIM. Among all clinical manifestations, Gottron's sign was significantly higher in DM patients than in ASS patients (*P* < 0.0001), while polyarthritis was more common in ASS patients (*P* = 0.026). Both groups had a high incidence of ILD (ASS, 80.0%; DM, 65.4%), and the incidence of respiratory infections (ASS, 30.0%; DM, 26.9%) and RP-ILD (ASS, 35.0%; DM, 34.6%) was similar in both groups. Of the 13 patients with respiratory infections, seven patients had a previous diagnosis of IIM, all on glucocorticoid therapy, one in combination with mycophenolate mofetil and two in combination with cyclophosphamide. Of all the 16 patients with RP-ILD, five were thought to suffer respiratory infections. Other clinical characteristics, myalgia, muscle weakness, mechanic's hands, and Raynaud phenomenon showed no significant differences.

### 3.2. Laboratory Data of the ASS and DM Patients

Compared with the normal reference values, most of the IIM patients had only mild to moderate elevation of creatine kinase (CK, 66.5 (26.0, 344.5)) and erythrocyte sedimentation rate (ESR, 24.0 (14.0, 36.0)), while most patients had normal C-reactive protein (CRP, 6.7 (4.7, 10.1)) levels (see Table 2). There was also no significant difference between the above indicators in ASS and DM patients. Most patients had mildly elevated lactate dehydrogenase (LDH, 293.5 (235.0, 448.0)) levels above the upper limit of normal reference values. The number of lymphocytes was significantly lower in DM patients than in ASS patients, while the number of white blood cells (WBC) and neutrophils did not differ significantly. In pulmonary function tests, both forced vital capacity (FVC) %, which reflects pulmonary restrictive ventilation dysfunction, and diffusing capacity of the lung for carbon monoxide (DLCO) %, which reflects diffusion function, were significantly lower in ASS patients than in DM patients. Data on myositis-specific antibodies were available for all IIM patients in our enrollment, and myositis-specific antibody types were abundant and representative of the disease groups in which they were located. Among them, the most frequent patients in the ASS group were with the anti-Jo-1 antibody, while in the DM group was the anti-MDA5 antibody, and notably, 26 of all 46 patients had a combination of the anti-Ro-52 antibody.

### 3.3. WBC, Lymphocytes, and Neutrophils in IIM Patients and Relationship with Lung Diseases

**Figure 1:**
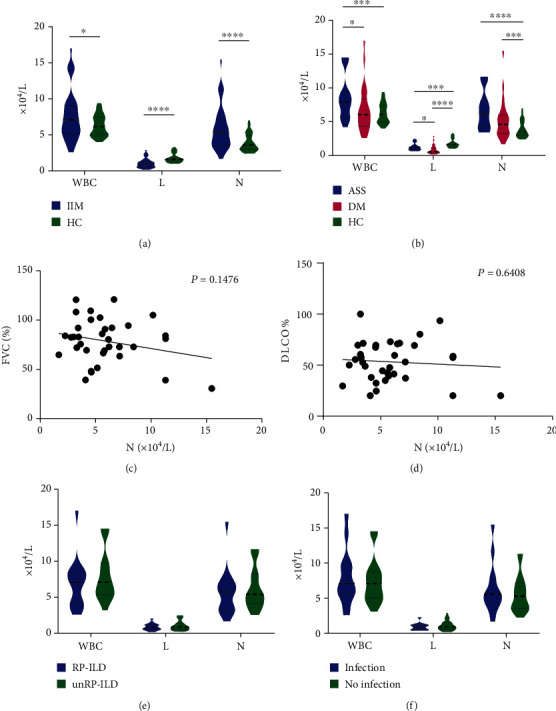
WBC, lymphocytes, and neutrophils in IIM patients and relationship with lung diseases. (a) Compared to healthy controls (HC), patients with IIM had significantly higher WBC counts (*P* = 0.023) and neutrophil counts (*P* < 0.0001), but lower lymphocyte counts (*P* < 0.0001). (b) ASS patients had higher WBC counts than healthy controls (*P* < 0.001) and DM patients (*P* = 0.073), while DM patients had lower lymphocyte counts compared to both ASS patients (*P* = 0.030) and healthy controls (*P* < 0.0001). ASS patients (*P* < 0.0001) and DM patients (*P* = 0.008) had higher neutrophil counts than healthy controls. (c, d) No correlations between neutrophils and FVC% and DLCO% were seen. (e) No differences in WBC, lymphocytes, and neutrophils between RP-ILD patients and other ILD patients. (f) No differences in WBC, lymphocytes, and neutrophils between the presence or absence of respiratory infections.

Compared to healthy controls (HC), patients with IIM had significantly higher WBC counts (*P* = 0.023) and neutrophil counts (*P* < 0.0001), but lower lymphocyte counts (*P* < 0.0001) (see Figure 1(a)). When subdivided, we found that ASS patients had higher WBC counts than healthy controls (*P* < 0.001), while DM patients had significantly lower lymphocyte counts compared to both ASS patients (*P* = 0.030) and healthy controls (*P* < 0.0001). Both ASS patients (*P* < 0.0001) and DM patients (*P* = 0.008) had higher neutrophil counts than healthy controls (see Figure 1(b)). The above immune cells were significantly different between IIM and healthy controls. However, when we performed correlation analysis with FVC% and DLCO%, indicators of lung function, there seemed to be no correlation between WBC, lymphocytes, and neutrophils and FVC% and DLCO% (see Figures 1(c) and 1(d) and Supplementary Figure 1). In addition, when the cell counts of RP-ILD patients (*n* = 16) were compared with those of other ILD patients (*n* = 17), we did not find any difference between the two groups (Figure 1(e)). It suggested that WBC, lymphocyte, and neutrophil counts, despite the differences between IIM and healthy controls, do not seem to be significantly suggestive of IIM-related lung diseases. Similarly, there was no clear relationship between the presence or absence of respiratory infections and the number of WBC, neutrophils, and lymphocytes in IIM patients (Figure 1(f)).

### 3.4. Decreased IL-6 Predicted RP-ILD of IIM Patients

**Figure 2:**
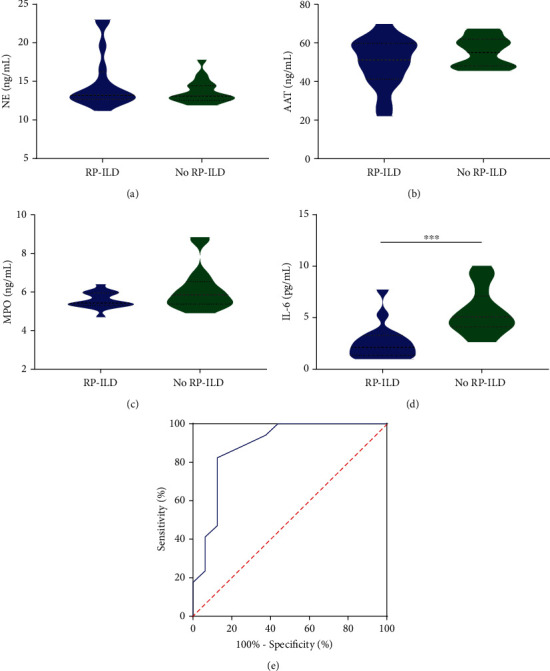
Decreased IL-6 predicted RP-ILD of IIM patients. (a–c) NE (*P* = 0.226), AAT (*P* = 0.138), and MPO (*P* = 0.067) did not differ significantly between patients in the RP-ILD and non-RP-ILD groups. (d) IL-6 (*P* = 0.0002) level was significantly lower in patients in the RP-ILD group. The results of the IL-6 assay ranged from 0.98 to 7.74 pg/mL (the second lowest was 1.31 pg/mL). (e) ROC analysis showed that the AUC values of IL-6 at 0.884 (95% CI 0.762–1.000, *P* = 0.0002).

Since neutrophil count did not appear to be directly related to lung diseases, we investigated some biomarkers associated with neutrophils. NE (*P* = 0.226) and MPO (*P* = 0.067) did not differ significantly between patients in the RP-ILD and non-RP-ILD groups; however, IL-6 (*P* = 0.0002) level was significantly lower in patients in the RP-ILD group (see Figures 2(a), 2(c), and 2(d)). There was also no significant difference in AAT between the two groups in the presence or absence of RP-ILD (*P* = 0.138) (see Figure 2(b)). ROC curves and their cut off values were also calculated. ROC analysis showed the AUC values of IL-6 (Figure 2(e)) at 0.884 (95% CI 0.762–1.000, *P* = 0.0002) with a cut off value 3.7 pg/mL, indicating decreased IL-6 to predict patients with RP-ILD.

### 3.5. MPO, NE, and IL-6 in Respiratory Infections of IIM Patients

**Figure 3:**
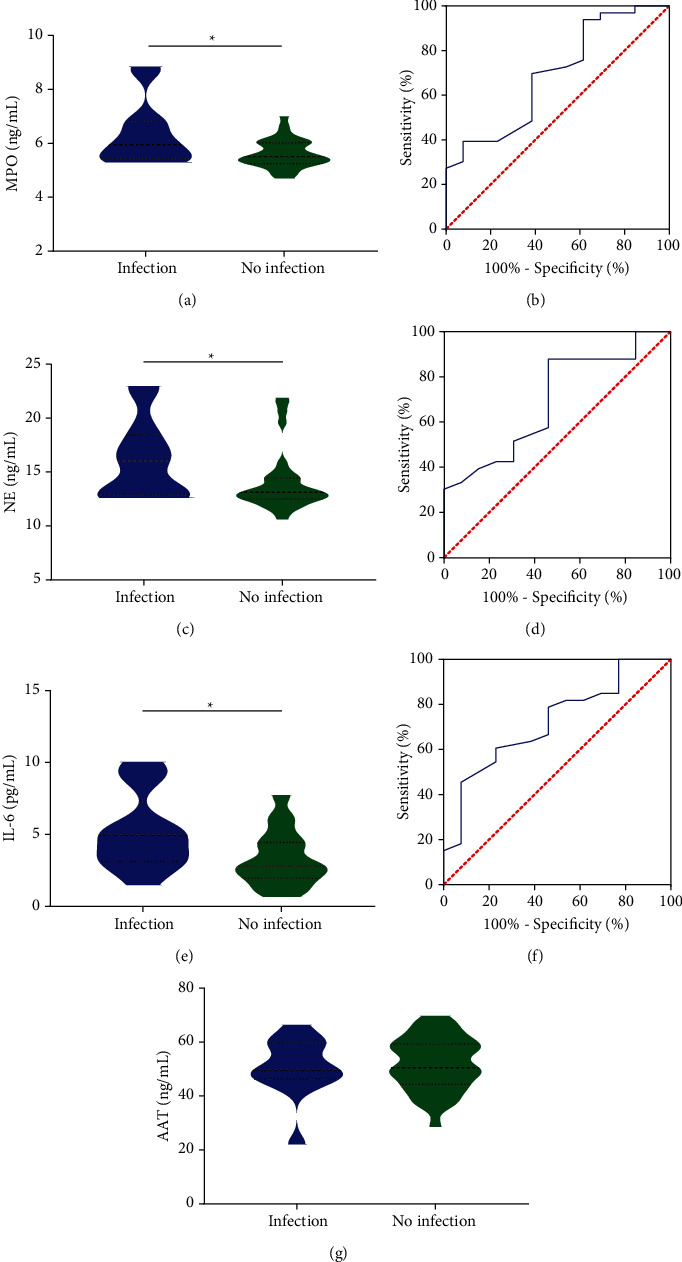
MPO, NE, and IL-6 in respiratory infections of IIM patients. In the ASS and DM patients with respiratory infections compared to patients without respiratory infections, MPO (a) (*P* = 0.049), NE (c) (*P* = 0.049), and IL-6 (e) (*P* = 0.014) were significantly higher. ROC analysis showed that the AUC values of MPO (b) at 0.692 (95% CI 0.523–0.861, *P* = 0.044), NE (d) at 0.692 (95% CI 0.5251–0.859, *P* = 0.044), and AUC values of IL-6 (f) at 0.720 (95% CI 0.561–0.880, *P* = 0.021). (g) AAT was not significantly different in the two groups of patients.

MPO (*P* = 0.049), NE (*P* = 0.049), and IL-6 (*P* = 0.014) were significantly higher in ASS and DM patients with respiratory infections compared to patients without respiratory infections (see Figures 3(a), 3(c), and 3(e)). We calculated ROC curves and their cutoff values. ROC analysis showed the AUC values of MPO (Figure 3(b)) at 0.692 (95% CI 0.523–0.861, *P* = 0.044); NE (Figure 3(d)) at 0.692 (95% CI 0.5251–0.859, *P* = 0.044); and AUC values of IL-6 (Figure 3(f)) at 0.720 (95% CI 0.561–0.880, *P* = 0.021). From the ROC curves, cut off values for MPO, NE, and IL-6 were 5.9 ng/mL, 15.95 ng/mL, and 2.71 pg/mL, respectively. Above results indicated a strong performance of MPO, NE, and IL-6 to identify patients with possible respiratory infections. There was no significant difference between AAT (*P* = 0.704) in patients with and without respiratory system infections (see Figure 3(g)).

### 3.6. AAT Might Be a Predictor of Poor Lung Function

**Figure 4:**
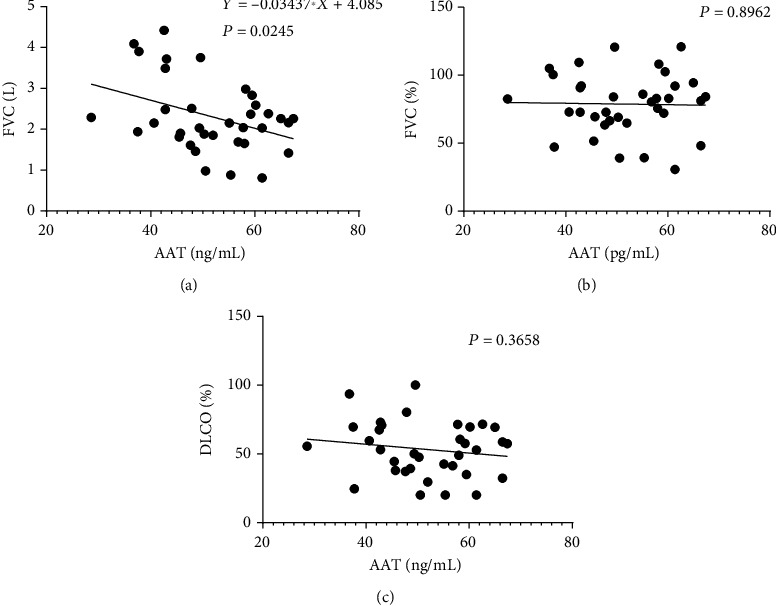
AAT might not be a good predictor of poor lung function. (a) There was a negative correlation between AAT and FVC (*r* = −0.034, *P* = 0.025). (b) No correlation of AAT with FVC% (*r* = −0.052, *P* = 0.896). (c) No correlation of AAT with DLCO% (*r* = −0.323, *P* = 0.366).

We found a negative correlation of AAT with FVC (*r* = −0.034, *P* = 0.025), Figure 4(a). But no correlation was seen in MPO, NE, or IL-6 (see Supplement Figure 2). However, AAT showed no correlation with FVC% (*r* = −0.052, *P* = 0.896) or DLCO% (*r* = −0.323, *P* = 0.366) (see Figures 4(b) and 4(c)). The correlation analysis of other indicators could be seen in Supplementary Figure 2.

## 4. Discussion

IIM is a serious autoimmune disease involving multiple organs, of which vascular inflammatory lesions of the skin, severe muscle involvement, and rapidly progressive interstitial lung disease are the most common reasons for hospitalization, and even death [18]. At the same time, treatment for active autoimmune diseases, including IIM, always depends on high doses of glucocorticoids and immunosuppressive agents. The therapy often exacerbates the patient's immunosuppressed state and brings infections, especially respiratory infections. Both progression of the IIM and respiratory infections may further contribute to RP-ILD. Therefore, early recognition and identification of respiratory infections and RP-ILD in IIM patients is crucial for rheumatologists, yet it is a major challenge to determine from imaging and common laboratory data.

A growing number of studies have identified biomarkers associated with IIM, particularly interstitial lung diseases. Bae and colleagues found the arylesterase and lactonase activities of paraoxonase-1 are significantly impaired in IIM patients and inversely associated with IIM disease activity and the presence of severe ILD [19]. Progranulin was also reported to be correlated with RP-ILD in anti-MDA5 antibody-positive DM patients [20]. Lian et al. developed a FLAIR Model with the metrics, ferritin, LDH, titer of anti-MDA5 antibody, chest HRCT score, and RP-ILD, which can predict the mortality risk of patients with anti-MDA5 antibody-positive DM [3]. As mentioned above, neutrophils, NETs, and their associated cytokines may all be involved in IIM-associated ILD, as well as being indicators of respiratory infections. Therefore, we investigated the role of these neutrophil-associated biomarkers in IIM-associated lung diseases, including respiratory infections, the development of RP-ILD, and lung function.

Although the concept of ASS has long been proposed, ASS has been considered part of DM until the new classification system in 2018. In the first part of our study, we summarize and compare the clinical and laboratory characteristics of patients with ASS and DM in this cohort. The general clinical features of IIM, including fever, myalgia, and muscle weakness, were present in both our patient groups, but there was no particular difference between the ASS and DM groups. The proportion of ASS patients with mechanic's hand and Raynaud phenomenon was higher than that of DM, but not statistically different. The most characteristic manifestations were Gottron's sign for DM and polyarthritis for ASS. Therefore, in addition to the myositis-specific and associated antibodies, Gottron's sign and polyarthritis provide us with a good means of differentiation when distinguishing between ASS and DM in clinical practice. The incidence of respiratory infections and RP-ILD was about 30% in both ASS and DM groups, with no significant difference. Although interstitial lung disease in DM is currently considered a major challenge for rheumatologists, the prevalence of ILD in ASS is indeed very high, up to 80%, and the incidence of RP-ILD is not lower than in DM.

CK, ESR, and CRP were essentially normal or mildly elevated in all patients in this cohort, and there were no significant differences between the ASS and DM groups. LDH was elevated in most patients, but there were no significant differences between the two groups. Lymphocytes were significantly lower in patients with DM, which has been discussed in previous literatures [21, 22]; however, WBC and neutrophils did not differ significantly. Interestingly, despite the seemingly more aggressive lung diseases in DM, pulmonary function tests suggested that both FVC% and DLCO% were significantly lower in patients in the ASS group than in those in the DM group in this cohort. Previous studies have identified poor lung function in patients with ASS and improvement of lung function after treatment [23–25]. Nevertheless, comparative studies of the differences in lung function between patients with ASS and those with DM are more lacking, which has led rheumatologists to pay more attention to lung involvement in ASS. All patients in the cohort had results for myositis-specific antibodies. Patients with ASS had various myositis-specific or associated antibodies, most often the anti-Jo-1 antibody, and the anti-MDA5 antibody was the most common antibody in DM patients.

When we introduced healthy controls for comparison, although the WBC, lymphocyte, and neutrophil counts were essentially normal or mildly elevated in patients with IIM, both the WBC and neutrophil counts were elevated, and the lymphocyte count was decreased compared to healthy controls. Subgroup analysis revealed that lymphocyte counts were lower in both ASS and DM patients than in healthy controls, while neutrophil counts were higher. These differences in cell counts suggest that lymphocytes and neutrophils are involved in IIM pathogenesis; however, their relationship with IIM lung disease still needs further discussion. Follow-up analyses revealed that none of the above cell counts was significantly suggestive of lung function, respiratory infections, or RP-ILD. Therefore, it is particularly important to explore the relevant biomarkers.

NETs is a newly discovered way of neutrophil death. Chromatin decondensation by citrullinated histone 3 (citH3) and granule proteins, including MPO and NE, is involved in the breakdown of membranaceous structures, allowing for a mixture of nuclear and cytoplasmic contents. Finally, cell-free DNA (cfDNA) and granule proteins are released as NETs [26]. Several studies have indicated that circulating NETs are potential biomarkers in many rheumatic diseases [27–29]. Circulating NETs were reported to be associated with higher levels of anti-MDA5 antibody and skin disease activity [30]. Low-density granulocytes and NETs containing LL-37 were also increased in patients with IIM and active disease and correlate with proinflammatory cytokines [8]. IIM NETs decreased the viability of myotubes in a citrullinated histone-dependent manner. Dysregulated neutrophil pathways may play pathogenic roles in IIM through their ability to directly injure muscle cells and other affected tissues [30]. There is a lack of research on the relationship between NETs and IIM-ILD, especially whether it could suggest RP-ILD. However, no significant differences in circulating NE and DNA were seen in our cohort of RP-ILD patients compared to those who did not present with RP-ILD. This suggests that although NETs may be involved in the pathogenesis of IIM, particularly in muscle and skin lesions, circulating NETs do not serve as important markers of RP-ILD pathogenesis. What surprised us, however, were the results for IL-6. Our data suggest that IL-6 was lower in IIM-ILD patients who did not present RP-ILD. There have been many studies on the relationship between IL-6 and IIM; serum IL-6 and immune cell expressions of IL-6 were increased in IIM patients than healthy people [31, 32]. Therefore, instead of simply comparing IL-6 with healthy individuals, we specifically studied IL-6 in RP-ILD. Studies have been found successful tocilizumab treatment of RP-ILD in anti-MDA5 antibody-positive DM patients [33]; we have also successfully treated an NXP-2 antibody-positive DM patient with tocilizumab [34]. The levels of serum IL-6 were reported to be higher in the RP-ILD subset than in the non-ILD subset or the chronic ILD subset in IIM, but there was no significance [35]. Our data suggest that IL-6 levels were significantly lower in patients with RP-ILD. This is contrary to the persistent perception that RP-ILD in IIM is closely associated with inflammatory cytokine storms like severe COVID-19 [36, 37], so it is cautious to assume that high circulating IL-6 was not a hallmark of RP-ILD, much less simply an indication for the utilization of tocilizumab, which is important for clinical practice. In addition, since pulmonary infection is one of the important triggers of RP-ILD, and IL-6 is significantly elevated in patients with respiratory infections, it is likely that some of the studies did not distinguish infection from RP-ILD well and therefore concluded that IL-6 is elevated in patients with RP-ILD.

Respiratory infections in IIM patients are also a frequent clinical situation and often need to be differentiated from ILD and even RP-ILD. Our study found that circulating NE, MPO, and IL-6 were significantly elevated in both ASS and DM patients with comorbid respiratory infections and have good diagnostic value. NETs and IL-6 have been studied extensively in pulmonary infections including COVID-19 [38, 39], and the findings are more consistent with the results of our study in concluding that circulating NETs and IL-6 could suggest respiratory infections. In particular, we have found that circulating IL-6 was decreased in patients with RP-ILD and significantly increased in respiratory infections. Although respiratory infections are an important predisposing factor for RP-ILD, the results of this study tell us very clearly that elevated IL-6 suggested infection rather than RP-ILD, and that the combined NE and MPO assays provide better clarity. This is of great importance for treatment decisions.

AAT is an important inhibitor of the protease, neutrophil elastase, which results in the breakdown of lung matrix elastin. And AAT deficiency (AATD) is a common reason for emphysema and thought to be an important factor in maintaining structural stability of the lung [40]. Our results found no clear implication of AAT for either RP-ILD or respiratory infections, while AAT showed a negative correlation with FVC in the study of lung function. However, when compared to FVC%, there was no significant correlation. We have a hypothesis that FVC declines reflect changes in lung structure, and AAT is important for maintaining the stability of lung structure, and the elevation of AAT may be the body's compensation for lung structure especially related to lung NE activation. Nevertheless, the comparison of FVC% is more practically meaningful than the absolute value of FVC, so AAT also does not seem to be a good predictor of lung function decline as a treatment. Perhaps expanding the sample size could make this conclusion clearer.

All in all, we have demonstrated that elevated IL-6 in serum is not a predictor of RP-ILD, elevated circulating NE, MPO, and IL-6 all suggest respiratory infections, and finally, AAT is an indicator of poor lung function in ASS and DM patients. Our observation is important, as it could provide information for better differentiating RP-ILD and respiratory infections. In addition, our study contributes to a more accurate understanding of the role of IL-6 in ASS and DM-related lung diseases. Elevated circulating IL-6 level does not imply the development of RP-ILD, but more likely represents respiratory infection, so it is inappropriate to apply IL-6 levels as an indication to initiate tocilizumab. Finally, elevated levels of circulating AAT, an important inhibitor of NE protease activity in lung tissue, predicted a decline in FVC, but it does not have a good predictive value for FVC% and therefore may not be a good predictor of lung function.

## Figures and Tables

**Table 1:** Demographic and clinical characteristics of the ASS and DM patients.

Variables	All	ASS	DM	*P* value
Numbers, *n* (%)	46 (100)	20 (43.5)	26 (56.5)	
Age, year	57.3 ± 10.9	61.1 ± 7.9	57.3 ± 12.2	0.061
Female, *n* (%)	33 (71.7)	17 (85.0)	16 (61.5)	0.08
Smoke, *n* (%)	9 (19.6)	3 (15.0)	6 (23.1)	0.494
Fever, *n* (%)	10 (21.7)	6 (30.0)	4 (15.4)	0.234
Myalgia, *n* (%)	11 (23.9)	4 (20.0)	7 (26.9)	0.585
Muscle weakness, *n* (%)	23 (50.0)	8 (40.0)	15 (57.7)	0.234
Gottron's sign, *n* (%)	33 (71.7)	9 (45.0)	24 (92.3)	<0.0001^∗∗∗∗^
Mechanic's hands, *n* (%)	12 (26.1)	7 (35.0)	5 (19.2)	0.227
Raynaud phenomenon, *n* (%)	7 (15.2)	5 (25.0)	2 (7.7)	0.105
Polyarthritis, *n* (%)	17 (37.0)	11 (55.0)	6 (23.1)	0.026^∗^
Respiratory infection, *n* (%)	13 (28.3)	6 (30.0)	7 (26.9)	0.818
Interstitial lung disease, *n* (%)	33 (71.7)	16 (80.0)	17 (65.4)	0.275
RP-ILD, *n* (%)	16 (34.8)	7 (35.0)	9 (34.6)	0.978

Values are mean ± SD*n* (%) unless specified otherwise. ^∗^*P* < 0.05 for comparison between ASS group vs. DM group using a *χ*^2^ test, Gottron's sign, and polyarthritis.

**Table 2:** Laboratory variants of the ASS and DM patients.

Variables	All	ASS	DM	*P* value
CK (U/L)	66.5 (26.0, 383.8)	63.5 (25.3, 344.5)	142 (33, 623.3)	0.209
ESR (mm/h)	24.0 (13.5, 36.3)	21.5 (15, 31.5)	28 (9.5, 37.3)	0.578
CRP (mg/L)	6.7 (4.7, 10.6)	8.1 (4.7, 10)	6.4 (4.5, 11.3)	0.324
LDH (U/L)	293.5 (232.8, 471.3)	285.0 (224.5, 337.5)	322.0 (258.0, 549.0)	0.333
WBC (×10^9^/L)	7.1 (5.2, 9.1)	7.9 (5.9, 9.4)	6.1 (4.4, 8.4)	0.073
Lymphocyte (×10^9^/L)	0.9 (0.5, 1.3)	1.2 (0.9, 1.5)	0.6 (0.4, 1.1)	0.03^∗^
Neutrophil (×10^9^/L)	5.3 (3.6, 7.2)	6.2 (4.2, 8.1)	4.6 (3.2, 6)	0.077
FVC (%)	81.2 (66.6, 92.2)	74.3 (64.8, 83.9)	84 (66.6, 105)	0.164
DLCO (%)	71.4 (48.9, 83.8)	48.3 (35.5, 58.4)	81.8 (77, 86.3)	<0.0001^∗∗∗∗^
Disease duration (month)	3.0 (2.4, 6.0)	3 (2.1, 6)	3 (2.8, 8.4)	0.401
Myositis antibodies, *n*				
Anti-Jo-1	13	13	0	
Anti-OJ	2	2	0	
Anti-EJ	4	4	0	
Anti-PL-7	1	1	0	
Anti-PL-12	3	3	0	
Anti-MDA5	12	0	12	
Anti-NXP-2	3	0	3	
Anti-TIF-1*γ*	4	0	4	
Anti-Mi-2	1	0	1	
Anti-Ro-52 (alone)	4	0	4	
Anti-Ro-52 (with other antibodies)	26	16	10	
Negative	2	0	2	

Values are mean ± SD*n* (%) or the quartile method. ^∗^*P* < 0.05 for comparison between ASS group vs. DM group using an independent sample *t* test or the Wilcoxon rank-sum test.

## Data Availability

The datasets used and/or analyzed during the current study are available from the corresponding author on reasonable request.

## Additional Points

*Key Messages*. (1) Decrease in IL-6 levels is a prognostic indicator for the development of RP-ILD. (2) Elevated circulating NE, MPO, and IL-6 all suggest respiratory infections.

## Supplementary Materials

Supplementary Materials Supplementary Figure 1: in all the patients with IIM: (A, B) no correlations between WBC and FVC% and DLCO% were seen. (C, D) No correlations between lymphocytes and FVC% and DLCO% were seen. Supplementary Figure 2: in all the patients with IIM: (A–C) IL-6 did not correlate with FVC%, FVC, or DLCO, respectively. (D–F) MPO did not correlate with FVC%, FVC, or DLCO, respectively. (G–I) NE did not correlate with FVC%, FVC, or DLCO, respectively.
