# Supplementary material for: Promising Neutrophil-Associated Biomarkers in Lung Diseases of Patients with Antisynthetase Syndrome and Dermatomyositis
Source: J Immunol Res. 2022 Sep 26;2022:1886083. doi: 10.1155/2022/1886083 (PMC9529515; doi:10.1155/2022/1886083)
Supplement: Supplementary Materials — Supplementary Figure 1: in all the patients with IIM: (A, B) no correlations between WBC and FVC% and DLCO% were seen. (C, D) No correlations between lymphocytes and FVC% and DLCO% were seen. Supplementary Figure 2: in all the patients with IIM: (A–C) IL-6 did not correlate with FVC%, FVC, or DLCO, respectively. (D–F) MPO did not correlate with FVC%, FVC, or DLCO, respectively. (G–I) NE did not correlate with FVC%, FVC, or DLCO, respectively. [file 1886083.f1.docx]

Supplementary Figure 1. In all the patients with IIM: (A, B) No correlations between WBC and FVC% and DLCO% were seen. (C, D) No correlations between lymphocytes and FVC% and DLCO% were seen.

Supplementary Figure 2. In all the patients with IIM: (A, B, C) IL-6 did not correlate with FVC%, FVC or DLCO, respectively. (D, E, F) MPO did not correlate with FVC%, FVC or DLCO, respectively. (G, H, I) NE did not correlate with FVC%, FVC or DLCO, respectively.
